# Validation of a simple-to-use, affordable, portable, wavefront aberrometry-based auto refractometer in the adult population: A prospective study

**DOI:** 10.1186/s12886-022-02684-5

**Published:** 2022-12-19

**Authors:** Divya Parthasarathy Rao, Kalpa Negiloni, Sivasundaravadivel Gurunathan, Selvaraj Velkumar, Anand Sivaraman, Adeeb Ulla Baig, B. Kumari, Kaushik Murali

**Affiliations:** 1 R&D, Remidio Innovative Solutions Inc., 11357 Nuckols Rd, #102, Glen Allen, Virginia, 23059 USA; 2 R&D, Remidio Innovative Solutions Pvt Ltd, Bengaluru, India; 3 Department of Pediatric Ophthalmology, Sankara Eye Hospital, Bengaluru, India

**Keywords:** Wavefront aberrometry, Auto refractometer, Auto refraction, Refractive error, InstaRef R20, Eye screening, Comprehensive eye examination

## Abstract

**Background:**

Refraction is one of the key components of a comprehensive eye examination. Auto refractometers that are reliable and affordable can be beneficial, especially in a low-resource community setting. The study aimed to validate the accuracy of a novel wave-front aberrometry-based auto refractometer, Instaref R20 against the open-field system and subjective refraction in an adult population.

**Methods:**

All the participants underwent a comprehensive eye examination including objective refraction, subjective acceptance, anterior and posterior segment evaluation. Refraction was performed without cycloplegia using WAM5500 open-field auto refractometer (OFAR) and Instaref R20, the study device. Agreement between both methods was evaluated using Bland-Altman analysis. The repeatability of the device based on three measurements in a subgroup of 40 adults was assessed.

**Results:**

The refractive error was measured in 132 participants (mean age,30.53 ± 9.36 years, 58.3% female). The paired mean difference of the refraction values of the study device against OFAR was − 0.13D for M, − 0.0002D (J0) and − 0.13D (J45) and against subjective refraction (SR) was − 0.09D (M), 0.06 (J0) and 0.03D (J45). The device agreed within +/− 0.50D of OFAR in 78% of eyes for M, 79% for J0 and 78% for J45. The device agreed within +/− 0.5D of SR values for M (84%), J0 (86%) and J45 (89%).

**Conclusion:**

This study found a good agreement between the measurements obtained with the portable autorefractor against open-field refractometer and SR values. It has a potential application in population-based community vision screening programs for refractive error correction without the need for highly trained personnel.

**Supplementary Information:**

The online version contains supplementary material available at 10.1186/s12886-022-02684-5.

## Background

With more than 150 million people affected globally, uncorrected refractive errors are the second leading cause of avoidable blindness and a major contributor to visual impairment [1]. Low- and middle-income nations like China and India account for the majority of such cases [2, 3]. There is an unprecedented need to develop tools to tackle this public health problem at scale.

A simple and economical solution for the correction of refractive errors is the use of glasses which are largely accepted [4]. Obtaining a prescription for glasses poses a substantial issue due to the shortage of qualified eyecare professionals [5]. Autorefractors have long been utilized in clinics to improve work-flow efficiency. However, majority of autorefractors are large, expensive, tabletop equipment with limited use for mass refractive error screening in environments with limited resources. In order to scale the solution to address this public health issue, novel devices leveraging on cutting-edge, user-friendly technology are much needed.

Adults have more stable refractive measurements with open field ARs, and subjective refraction is the gold standard for patient acceptance. In recent times, a few portable automated devices namely QuickSee Flip/e-see (PlenOptika/Aurolab), NETRA (EyeNetra), and SVOne (Smart Vision Labs), that might be useful in low-income populations, have been developed [6–9]. InstaRef R20, is one such device which is based on Shack Hartmann aberrometer technology with several advantages. It is a low weight (400 g), simple to use device and measures refractive error of good range (− 10.0 D to + 10.0D sphere and − 5.0D to + 5.0D of cylinder) [10]. It allows patients to look at far distance relaxing accommodation and has a tilt warning system. It offers an easy-to-use interface, the ability to print findings immediately, and a desktop program for managing patient data. This hand-held, affordable (fraction of the cost of a tabletop device) autorefractor could potentially help reduce the burden of this problem by increasing access to refractive error screening, especially in low-resource settings.

In this study, we aimed to validate the accuracy of this novel hand-held, portable autorefractor for measuring refractive errors by comparing against subjective refraction and open-field autorefractor in light of promising results from pilot trials. We performed non-cycloplegic refraction measurement on adult subjects using standard methods and study device and evaluated their agreement.

## Methodology

A prospective, cross-sectional study was conducted at a tertiary eyecare center, Sankara Eye Hospital, Bengaluru, South India between 10th October and 31st October 2021. The study was approved by the Institutional Ethics Committee, Sankara Eye Hospital, Bengaluru, India (Approval number: SEH/BLR/EC/2O21/47) and adhered to the tenets of the Declaration of Helsinki. Written informed consent was obtained from all the adults who participated in the study.

### Inclusion and exclusion criteria

The study included subjects older than 18 years with best corrected visual acuity (BCVA) of 20/20 in each eye and refractive errors between Sph+/−10D, Cyl +/−5D. Subjects with significant media opacity- corneal opacity, advanced cataract, vitreous hemorrhage, and posterior segment pathology affecting visual acuity were excluded from this study. Subjects with amblyopia, history of recent eye surgery within the past 2 weeks, active eye infection/ inflammation were not allowed to participate. Those who were on medication interfering with visual acuity, unstable medical condition or deemed unfit by the investigator and pregnant women were also not enrolled.

All subjects underwent a refractive error measurement on both eyes followed by a thorough ophthalmic examination of the anterior and posterior segment. At first, uncorrected visual acuity (UCVA) was measured using Snellen chart at 6 m under appropriate light conditions. Following objective retinoscopy, subjective refraction was performed and the BCVA was recorded. Refractive error was measured using Grand Seiko WAM5500 open field auto refractometer (OFAR).

**Fig. 1:**
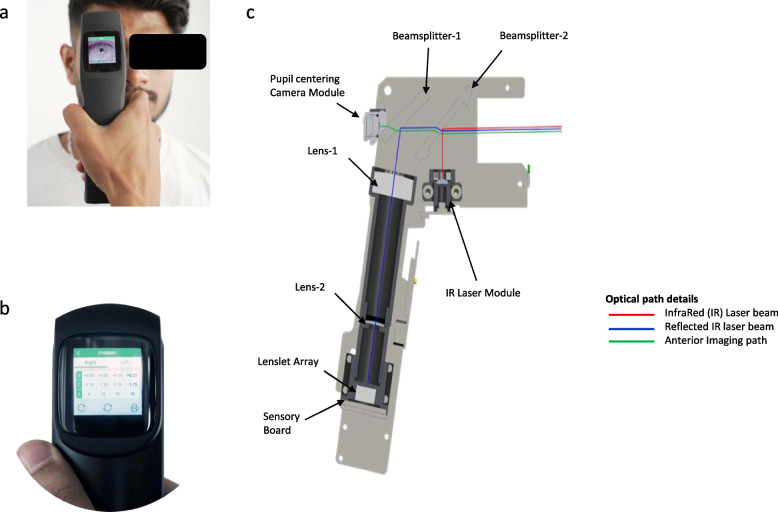
**a** Refractive error measurement using InstaRef R20, portable wavefront aberrometry-based auto refractometer. The subject is looking at far fixation distance using the other open eye (**b**) Refractive error values on the study device screen with printable or easy data transfer (**c**) Design and optical path of InstaRef R20

Next, a minimally trained optometrist performed a measurement using the handheld study device InstaRef R20 (Remidio Innovative Solutions Pvt. Ltd., Bengaluru, India) ensuring good alignment. The measurement of refractive error using the study device is shown in Fig. 1(a & b). Proper alignment was ensured by holding the device at the subject’s eye level and the crosshair on the device screen was centered to the pupil. Right eye measurement was taken with the patient being instructed to view the external visual target with the left eye kept open. The device took three readings and provided a final averaged value. The same process was repeated for the left eye.

### About study device

InstaRef is a wavefront aberrometer-based auto refractometer that allows monocular measurements with the subject fixating at a far distance through the other eye, relaxing the accommodation. The optics of the system are described in Fig. 1c. In this technique, a point on the retina is illuminated using an Infrared (IR) beam (in red) of 850 nm from a laser module. The reflected IR laser beam (in blue) passes through the lens system. The wavefront sensor consists of a microlenslet array (12.92 X 8.75 mm and 1.55 mm thickness) that constrains the remitted light to a pattern of spots that are detected with an image sensor (4.76 X 5.61 mm). The tilt of the wavefront part that enters each lenslet is directly correlated with the location of each spot on the image sensor. The spot positions will aid in calculating the phase of the wavefront which is related to the ocular aberrations. The location of each light spot is compared to the flat/non-aberrated wavefront. The average slope of each wavefront is computed. These spot-position coordinates are then used in modal reconstruction to approximate the three-dimensional wavefront topology for lower-order aberrations using standard mathematical calculations. The device also includes a pupil-centering module and an audible tilt warning system. It can measure in a pupil of a minimum of 3 mm diameter.

All the refraction measurements were transformed using Fourier vector decomposition to spherical and cylindrical scale values using the below formulae. The spherical equivalent was calculated as sphere value + cylindrical value/2. According to vector principles, astigmatism has two orthogonal vectors known as J0 and J45. J0 is the horizontal and vertical component of astigmatism, and J45 is the oblique component of astigmatism. The following equations were used to calculate the vectors:

$$\textrm{J}0=\left(-\textrm{C}/2\right)\ \cos \left(2\textrm{a}\right),\textrm{J}45=\left(-\textrm{C}/2\right)\ \sin \left(2\textrm{a}\right)\ \textrm{where}\ \textrm{C}=\textrm{cylinder}\ \textrm{power},\textrm{a}=\textrm{axis}\ \textrm{of}\ \textrm{astigmatism}$$

The agreement between the study device, subjective refraction and OFAR measurements was evaluated using a Bland-Altman analysis with 95% limits of agreement (LOA) on each power vector component (M, J0, and J45). The bias between the methods was compared. Agreement within thresholds of 0.25D and 0.5 D for M, J0, and J45 was evaluated. The repeatability of the study device was also determined by measuring the refractive error three times in a subset of 40 patients. Intraclass correlation coefficient (ICC) was calculated and values below 0.5 were considered poor, between 0.5 and 0.75 as moderate, between 0.75 and 0.9 as good and any value above 0.9 as excellent repeatability [11]. Any *p-value* < 0.05 was considered statistically significant. The minimum sample size calculated was 132 subjects based on a relative precision of 10%, having 90% power and a 95% confidence level. All data were entered, and statistical analysis was performed using Microsoft Excel and XLSTAT 2022.

## Results

A total of 132 adult subjects with an average age of 30.53 ± 9.36 years were included. No patients were excluded following enrollment. 58.3% were females (*n* = 77, 29.38 ± 9.68 years) and 41.7% were males (*n* = 55, 32.15 ± 8.71 years). Refractive error measurement data obtained by different methods were used to derive M, J0 and J45 values in diopter (Table 1). No significant difference was observed in the right and left eyes for the measured values of the sphere, M, J0 and J45. Additionally, the data was highly correlated between the right and left eye. Therefore, further analysis was performed on the right eye readings only.

**Table 1:** Average values of refractive outcomes (M, J0 and J45) measured using InstaRef R20, Open-field Auto Refractometer and Subjective Refraction

Methods of measurement	Sphere (D) Mean ± SD (95% CI)	Cylinder(D) Mean ± SD (95% CI)	M* (D) Mean ± SD (95% CI)	J0** (D) Mean ± SD (95% CI)	J45** (D) Mean ± SD (95% CI)
**InstaRef R20**	−0.66 ± 1.64 (− 0.94 to − 0.38)	−0.91 ± 0.75 (− 1.04 to − 0.78)	− 1.10 ± 1.72 (− 1.40 to − 0.81)	− 0.06 ± 0.28 (− 0.11 to − 0.01)	0.14 ± 0.49 (0.06 to 0.22)
**Open-field Auto refractometer**	− 0.80 ± 1.86 (− 1.12 to − 0.49)	− 0.93 ± 0.76 (− 1.06 to − 0.80)	− 1.24 ± 1.90 (− 1.56 to − 0.91)	−0.06 ± 0.41 (− 0.13 to 0.01)	0.01 ± 0.41 (− 0.06 to 0.08)
**Subjective Refraction**	−0.88 ± 1.78 (− 1.18 to − 0.58)	−0.86 ± 0.72 (− 0.98 to − 0.73)	− 1.19 ± 1.88 (− 1.5 to − 0.87)	0.00 ± 0.27 (− 0.04 to 0.05)	0.17 ± 0.35 (0.11 to 0.23)

*M- Spherical Equivalent, **J0 and J45 – Cylindrical components

### InstaRef R20 vs open field auto refractometer (OFAR)

**Fig. 2:**
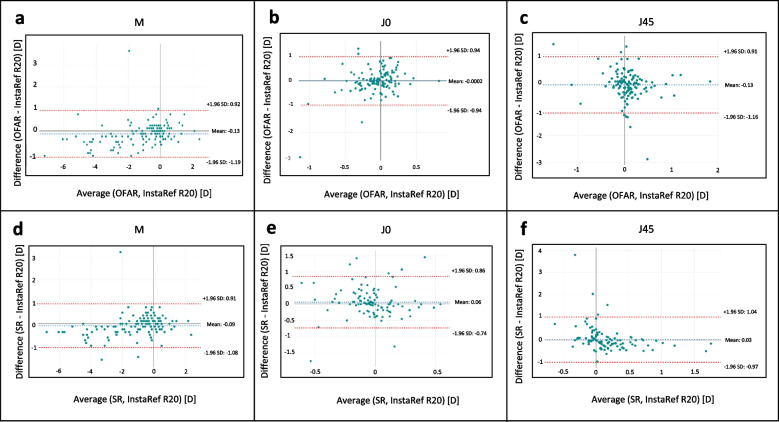
Bland-Altman plots showing bias and 95% limit of agreement. **a**, **b** and **c** - agreement between InstaRef R20 and Open-field auto refractometer (OFAR) for M, J0 and J45 vectors respectively. **d**, **e** and **f** - agreement between InstaRef R20 and subjective refraction (SR) for M, J0 and J45 vectors respectively

The mean values of Sphere, Cylinder, M, J0 and J45 of the study device and OFAR are presented in Table 1. The average M readings were slightly more myopic with OFAR (M = − 1.24D) when compared to InstraRef R20 (M = − 1.10D). A statistically significant difference was observed for mean values of M (mean paired difference of − 0.13D, *p* = 0.02) and J45 (mean paired difference of − 0.13D *p* = 0.025) between InstaRef R20 and OFAR. There was no statistically significant difference in J0 values. However, clinically, the mean paired difference for M, J0 and J45 were small. The device agreed within 0.5D of OFAR values for M (78%), J0 (79%) and J45 (78%). Table 2 shows the mean paired differences and proportion of values within +/− 0.25D and +/− 0.50 D for M, J0 and J45 values between the study device and OFAR. To compare the difference between each of the findings and OFAR, the 95% limits of agreement (LOA) was quantified using the Bland and Altman method. Here, the difference between each vector measurement against OFAR was determined, and the LOA was calculated as 1.96 multiplied by the SD of the differences. These values are shown in Table 2, with lower values representing better agreement. Bland-Altman plots between the study device and OFAR are shown in Fig. 2 (a, b, c) for M, J0 and J45 vectors, respectively.

**Table 2:** Paired mean difference and 95% limit of agreement (LOA) for M, J0 and J45 values of Subjective refraction, and Open-field AR when compared to InstaRef R20

Methods of measurement	Paired mean difference	***p-value***^**a**^	95% LOA	Agreement within +/− 0.25D	Agreement within +/− 0.50D
	**M**				
OFAR	−0.13	0.022	−1.19 to 0.92	55%	78%
SR	−0.09	0.195	−1.08 to 0.91	61%	84%
	**J0**				
OFAR	−0.0002	> 0.99	− 0.94 to 0.94	55%	79%
SR	0.06	0.315	−0.74 to 0.86	67%	86%
	**J45**				
OFAR	−0.13	0.025	−1.16 to 0.91	52%	78%
SR	0.03	0.934	−0.97 to 1.04	67%	89%

*p-value*^a^ obtained by Paired t-test, Subjective refraction (SR), and Open-field AR (OFAR)

### InstaRef R20 vs subjective refraction (SR)

The mean paired difference between study device and SR for M, J0 and J45 were not found to be statistically or clinically significant. The device agreed within 0.5D of SR values in 84% for M, in 86% for J0 and in 89% for J45. Similarly, OFAR agreed within 0.5D of SR in 82% for M, 83% for J0 and 87% for J45. Table 2 shows the mean paired differences and proportion of values within +/− 0.25D, +/− 0.50D for M, J0 and J45 values between the study device and SR. There was no statistically significant difference in M (0.05 ± 0.45, *p* = 0.236) and J0 values (0.06 ± 0.45, *p* = 0.122) between SR and OFAR and J45 were significantly different (0.16 ± 0.49, *p* < 0.01). However, clinically the differences were small and acceptable. Bland-Altman plots between the study device and SR are shown in Fig. 2 (d, e, f) for M, J0 and J45 vectors, respectively. All the paired mean differences between the different measurements remained mostly within the range of + 1/− 1 D (±2 SD).

In a sub-group analysis, comparing the study device against OFAR and SR based on the type of refractive error (within +/− 0.50DS (*n* = 58), Myopia < − 3.00D (*n* = 39), myopia > − 3.00D (*n* = 25) and Hyperopia(*n* = 10)), the mean paired difference was clinically insignificant (<+/− 0.50D) for M, J0 and J45 values. A similar clinically insignificant difference (< +/− 0.50) in mean paired difference for all the vector values was noted based on age categorization as well (18 to 30 years (*n* = 70), 31 to 40 years (n = 39) and ≥ 40 years (*n* = 23) group).

### Repeatability of InstRef R20

The refraction of 40 subjects was repeated three times and the ICC was found to be above 0.88 for Sphere, Cylinder, Axis and M values. Table 3 summarizes the repeatability analysis of the device using the Intra-class correlation (ICC) test on three consecutive readings.

**Table 3:** Repeatability analysis of three consecutive readings of InstaRef R20 using Intra-class correlation (ICC) test

Parameters	Mean ± SD^**a**^	***p-value***^**b**^	ICC^**c**^	95% confidence interval
Sphere	0.02 ± 0.21	0.984	0.988	0.979–0.993
Cylinder	0.04 ± 0.13	0.904	0.985	0.975–0.992
Axis	11 ± 6.47	0.593	0.885	0.806–0.935
Spherical Equivalent (M)	0.04 ± 0.20	0.986	0.988	0.980–0.993

^a^ Mean and standard deviation (SD) of the differences between each of the three repeated measures, ^b^ Repeated Measures ANOVA, ^c^ Two-way model, absolute agreement average measures

## Discussion

This prospective study on adult subjects compared a new hand-held, portable, Shack-Hartmann aberrometry-based autorefractor, against a validated objective measurement tool (open-field autorefractor) and subjective refraction. It was found to have a good agreement and the differences were within the clinically acceptable limits.

A portable auto refractometer which is reliable and simple to use is the need of the hour for large-scale refractive error screening. Gold standard retinoscopy is not only cumbersome but also practically difficult to perform in outreach settings in bright daylight as reflexes are not visualized. Additionally, it requires experienced eyecare professionals who are in acute shortage in rural areas. Most of the currently available auto refractometers are not only expensive but also not suitable for field use as they are not portable. The spherical equivalent and cylindrical values of wavefront-aberrometry-based handheld, portable auto refractometers are validated against other techniques in previous studies [6–9, 12, 13]. Padhy et al. compared different auto refractometers (rotary prism-based closed field, photorefraction-based spot screener and wavefront-based device) against standard retinoscopy and reported it comparable and among them, wavefront performed better in all the measured parameters [14]. Table 4 summarizes the comparison of wavefront-based auto refractometers against other techniques.

**Table 4:** Comparison of different portable, handheld wavefront-aberrometry-based auto refractometers (pre cycloplegic)

Study	Ciuffreda & Rosenfield, 2015 [8]	Jeganathan, Woodward et al, 2018 [ 7]	Rubio et al, 2019 [6]	Current study
**Device compared**	SVOne vs Retinoscopy	Netra, EyeNetra vs Retinoscopy	QuickSee Flip/e-see vs Nidek ARK1	InstaRef R20 vs WAM5500 open field
**Comparative Technique**	Knife edge	Knife edge	Scheiner’s double pinhole	Open-field infrared binocular
**Sample**	50	152	54	132
**Age (years)**	18–31	20–90	22–65	18–52
**Wavefront device vs retinoscopy - Mean Difference (95% LOA)**				
**M**	0.48^a^	−0.27 (range − 2.38, 3.00)	0.02 ± 0.40	−0.13 (−1.19 to 0.92)
**J0**	0.09^a^	0.11 (range − 2.00, 2.50)	−0.04 ± 0.15	−0.0002 (− 0.94 to 0.94)
**J45**	0.01^a^		0.01 ± 0.10	−0.13 (−1.16 to 0.91)
**Wavefront device vs Subjective refraction- Mean Difference (95% LOA)**				
**M**	−0.43 (−1.3 to 0.45)	–	0.09 ± 0.39	−0.09 (− 1.08 to 0.91)
**J0**	− 0.20 (− 0.70 to 0.45)	–	− 0.06 ± 0.13	0.06 (− 0.74 to 0.86)
**J45**	0.05 (− 0.35 to 0.38)	–	0.02 ± 0.12	0.03 (− 0.97 to 1.03)

^a^ Difference in mean values of SVOne and Retinoscopy values

OFAR eliminates accommodation as the device allows the patient to view far distances. It is reported to be reliable and repeatable in both adult and pediatric groups [15]. In comparison against this validated OFAR, the mean paired difference of M, J0 and J45 values were small (− 0.13, − 0.0002 and 0.13 respectively). The device agreed within 0.5D for M (78%), J0 (79%) and J45 (78%). Ciuffreda and Rosenfield validated SVOne, a smartphone-based auto refractometer against standard subjective and objective refraction and found no significant differences in the measurements similar to the current study and recommended its use in optometry clinics and vision screening [8]. An interesting aspect highlighted in the study was the effect of tilt of the instrument on astigmatic values. Tilting the instrument by 5 degrees had little impact and greater than 10 degrees showed that the Hartmann-shack images were not clearly visible posing errors on astigmatic values. The current study device has infrared imaging feedback that ensures pupil centration. Along with a built-in audible tilt warning system, it allows for high accuracy of cylindrical values. A similar portable wavefront-based auto refractometer validated against retinoscopy & SR in adults found comparable results and proposed its application in population settings with limited access to eye care professionals [7].

In a review of portable wavefront aberrometry-based autorefractors, it was emphasized that the accuracy of the device is well understood when compared to the gold standard technique and subjective acceptance [16]. In comparison to subjective refraction, the differences were statistically insignificant for M, J0 and J45 values. The device agreed within 0.5D in more than a majority of the study cohort- 84% for M, 86% for J0 and 89% for J45. This was similar to OFAR when it was compared against SR with an agreement within 0.5D of 82% for M, 83% for Jo and 87% for J45. Supplementary Table 1 presents different studies that have compared the spherical and cylindrical values of open-field AR against subjective refraction and its agreement within 0.25D and 0.50D thresholds [9, 15]. The proportion of values of the study device against subjective refraction were comparable if not better to the previous studies with > 61% within 0.25D and > 84% within 0.5D difference.

Recent studies comparing wavefront autorefractors and subjective refraction in adults (summarized in Table 4) have found average differences in spherical equivalent measurements and cylindrical components higher than the current device [6–9]. This comparison is pivotal in using the refraction values as a starting point for subjective refraction and prescribing refractive error correction. It is also valuable in large population-based studies where healthcare nurses, technicians and others are trained for primary level eye screening instead of optometrists and ophthalmologists. Although retinoscopy and subjective refraction still remain the gold standard method for prescribing refractive error correction, new innovative devices such as InstaRef R20 can serve as a quick and reliable tool, especially in resource constrained settings.

In a subgroup analysis on the type of refractive error in the current study, no clinically significant difference was noted between the study device when compared against OFAR and SR for M, J0 and J45. There was a good distribution of sample size from +/− 0.5D to > − 3D myopia. There was no significant difference when compared against different age groups.

The sources of error can be classified into subject-related, operator-related and instrument-related errors. While the study device prevents instrument-induced myopia to a large extent given the open-field nature, there is still a possibility of few errors due to a strong accommodation in young adults. Variations in pupil size variation can impact refraction values. Errors can be minimized by conducting objective wavefront readings and subjective acceptance in a single lighting condition. Operator-related errors can be reduced with training. This will ensure no head tilt of the subject, adequate alignment of the cross-hairs at the pupil center, correcting alignment when audible feedback is heard from the tilt warning system. There are few instrument-related errors that need to be considered as well. The average Spherical equivalent power error we found was − 0.08D. The next step would be to reduce this systematic error by measuring the refraction in a large number of eyes and incorporating the offset into the calibration. Further optimization comprises of minimization of the wavefront reconstruction error and/or the number and sampling position of the microarray lenslets to improve the accuracy. The calculation of lower-order aberration needs an accurate estimation of the mean values of the point signal intensities that can otherwise contribute to instrument-induced errors as well.

There are a few limitations of the study that need to be considered. One is the number of participants in some refractive error subgroups (hyperopes) was small and hence difficult to draw definitive conclusions. Secondly, the age-group range was 18–52 years. Understanding whether the performance of the device is upheld even in the elderly above 60 years with smaller pupils is yet to be determined.

The study results indicate that the device can be used in clinical or community screening settings as a part of comprehensive eye examination or screening. The advantage of the device is that it is a low-cost, portable, simple to use tool and allows integration into an electronic medical record (EMR) system. Additionally, this tool can help decentralize screening closer to patient context, enable point-of-care testing even at home, and cater to special needs groups such as wheelchair-bound patients.

## Conclusion

The study results indicate that the InstaRef R20 wavefront-based portable auto refractometer provides refractive error measurements in the adult population that are not clinically significantly different from the open-field and subjective refraction values. This device would further serve beneficial in low-resource settings without the need for highly trained personnel which can improve eye care in developing countries. The wavefront aberrometry-based auto refractometer can also be used as a quick, portable, affordable and reliable device for refractive error measurements in large population-based eye screening programs as well as an adjunct in the standard optometric examination.

## Supplementary Information

**Additional file 1.** Table S1. Agreement between refractive parameters measured by Objective Refraction against Subjective Refraction

## Data Availability

The data cannot be shared publicly because of sensitive patient information, but are available on request to the corresponding author.
