# Supplementary material for: Validation of a simple-to-use, affordable, portable, wavefront aberrometry-based auto refractometer in the adult population: A prospective study
Source: BMC Ophthalmol. 2022 Dec 19;22:498. doi: 10.1186/s12886-022-02684-5 (PMC9764520; doi:10.1186/s12886-022-02684-5)
Supplement: Supplementary file 1 — Additional file 1. Table S1. Agreement between refractive parameters measured by Objective Refraction against Subjective Refraction [file 12886_2022_2684_MOESM1_ESM.pdf]

Supplementary Table 1: Agreement between refractive parameters measured by Objective Refraction against Subjective Refraction

Refractive parameters	Instaref R20 Vs SR (Current study)		OFAR vs SR (Current study)		OFAR vs SR (Sheppard et al[15])		OFAR vs SR (Durr et al[9])	
	≤0.25D	≤0.5D	≤0.25D	≤0.5D	≤0.25D	≤0.5D	≤0.25D	≤0.5D
**M**	61%	84%	53%	82%	57%	73%	63%	89%
**J0**	67%	86%	55%	83%	-	-	-	-
**J45**	67%	89%	60%	87%	-	-	-	-
**Cyl**	**-**	**-**	-	-	74%	93%	82%	98%

SR: Subjective Refraction, OFAR: Open-field auto refractometer, M: Spherical Equivalent, J0 and J45- Cylindrical components, Cyl – Cylinder
